# PAIA assays: A new bead-based assay system for high throughput protein quantification

**DOI:** 10.1186/1753-6561-9-S9-P62

**Published:** 2015-12-14

**Authors:** Christine I Wosnitza, Kristina Lechner, Sebastian Giehring

**Affiliations:** 1 PAIA Biotech GmbH, 51105 Cologne, Germany

## Background

Key words: protein quantification / cell line development / IgG assay kit / high producer screening / high throughput

**Figure 1:**
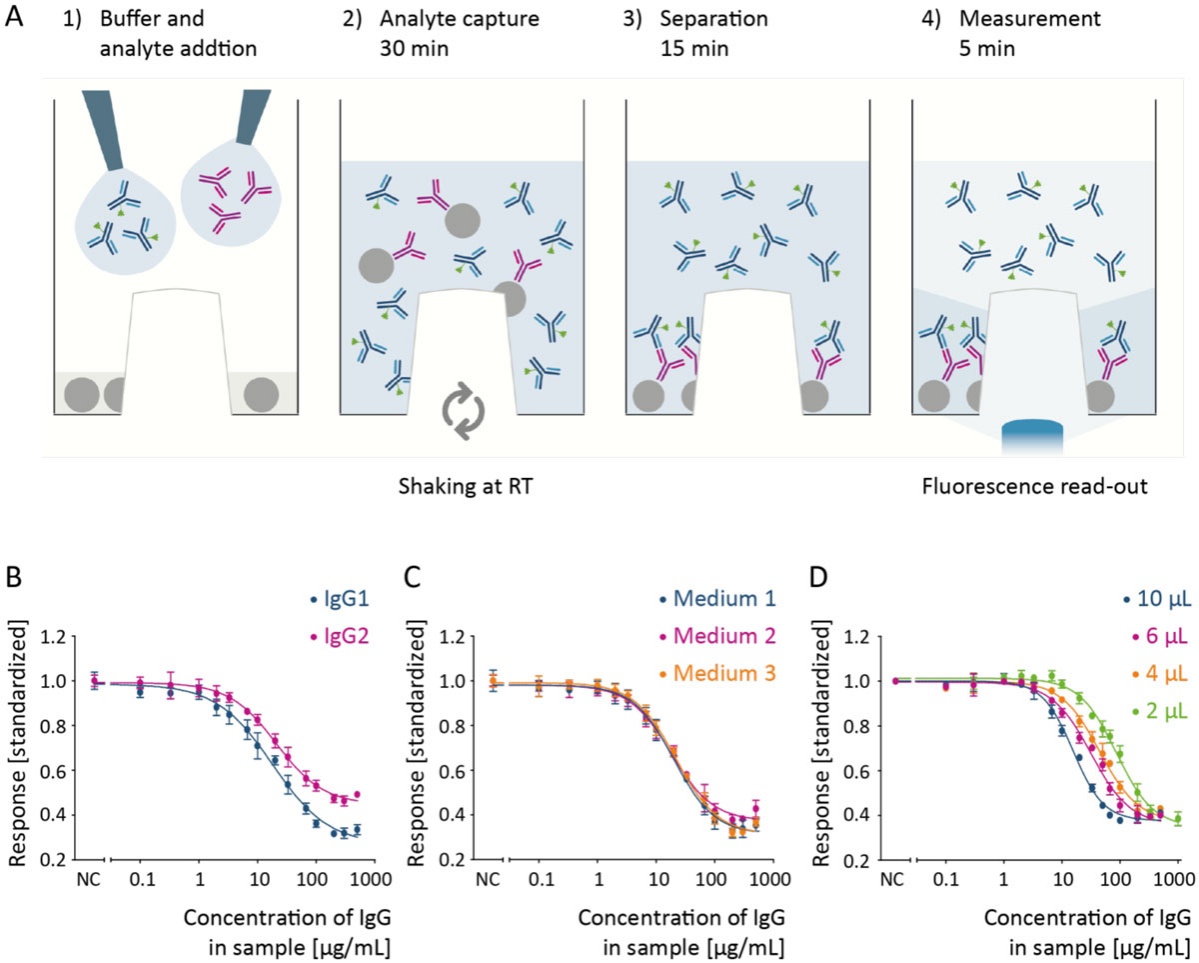
**PAIA Human IgG Fc/Fab assay**.(a) Description of the PAIA assay workflow: Each of the wells of the 384 well PAIA plate contains pre-dispensed dried capture beads. The depicted procedure is run in parallel in each of the wells. The unique structure of the plate allows detection of the unbound fluorescence marker only through the transparent protrusion which keeps the beads outside the detection zone and light path. (b) IgG1 and IgG2 quantification in buffer. (c) IgG1 quantification in cell culture media. It is recommended to prepare standards and samples in the same matrix. Medium 1: Gibco Freestyle CHO + 8 mM Glutamax, Medium 2: BD Select CHO + 8 mM L-Ala-L-Gln, Medium3: Biochrome ISF-1 + 10% FBS. (d) IgG1 calibration curves for several sample volumes. The dynamic range is adapted to higher (2 μL) or lower (10 μL) IgG concentrations by the variation of sample volume.

The patent pending PAIA technology provides a novel platform for the quantification of proteins, e.g. monoclonal antibodies. PAIA assays consist of functionalized beads to capture the protein of interest (the analyte) and a fluorescent marker to detect the analyte. In contrast to other bead-based assays, which measure the fluorescence intensity of marker that is bound to the beads [1], PAIA assays measure the fluorescence of unbound marker remaining in solution. We use protrusions on the bottom of the microplate that separate captured analyte-marker complexes from unbound fluorescent marker. Therefore, no washing steps are needed and the assay can be performed in an automation-friendly 384-well plate format (Figure 1A).The time to result, the amount of sample required, and the hands-on time are substantially reduced compared to other immunoassay formats. Hence, this novel approach is particularly suitable for high throughput analysis of protein containing samples like supernatants of antibody producing cells.

## Material and methods

The PAIA platform is based on 384-well plates and all assays were performed in a volume of 60 µL. If not stated otherwise, a sample volume of 6 µL was used. The sample as well as fluorescence marker in reaction buffer were added to the microplate which contained dried capture beads. After addition of buffer and sample, the microplate was incubated for 30minutesat 2000 rpm on an orbital shaker at room temperature and subsequently allowed to stand for 10-15 minutes. The read-out is obtained with either a plate reader or a fluorescence microscope (SynenTec, Elmshorn, Germany) in less than five minutes per plate. The data depicted in Figure 1D was obtained by incubating the assay on the BioShakeXP (Q.Instruments, Jena, Germany) at 1800 rpm for 40 minutes, followed by 5 minutes at 1000 rpm and 10 minutes without agitation. The protrusion on the microplate bottom ensures that the beads are settling outside the detection window. Hence, only the unbound fluorescent marker was detected when measuring on a fluorescence plate reader in bottom read modus at 485/520 nm (Safire, Tecan, Männedorf, Switzerland).Calibration curves were calculated in the PAIA evaluation software with a 4-parameter fit.

## Results and discussion

PAIA assays offer the possibility to choose a marker against the specific epitope of the analyte that shall be detected. For example, in a PAIA assay to quantify human IgG1, Protein A beads capture the Fc part of IgG and the fluorescent marker can be directed either against the whole antibody or against specific regions like the Fab fragment or the light chain. The PAIA Human IgG Fc/Fab assay kit uses a marker targeting the Fab region of human IgG. Due to the Protein A capture, the assay is suitable for IgG1, IgG2 (Figure 1B) and IgG4 (data not shown). The curves reflect different affinities of Protein A and fluorescence marker to the IgGs. In addition, the IgG assay was tested for influences of cell culture media and supernatants of HEK and CHO cells. The raw data signal was slightly elevated in most samples. However, when the calibration standards are prepared in the same matrix as the samples almost no difference is detected (Figure 1C). The performance values of this assay are displayed in Table 1. An IgG1 concentration range of about 5 - 200 μg/mL is covered in the assay when using 6 µL sample. Variation of the sample volume allows adjustment of the dynamic range to the expected analyte concentration (Figure 1D). One plate can be processed in an hour; the throughput can be increased by the use of multiple shakers in parallel. Due to the standardized plate format and the simple mix-and-measure protocol the assay can be easily run in an automated setup.

**Table 1:** Performance values of the Human IgG Fc/Fab assay

Sample volume	6 μL
Assay volume	60 μL
IgG concentration	5 - 200 μg/mL
Limit of detection	~ 5 μg/mL
CV	~ 10%

## Conclusions

We believe that the high throughput, the small sample volume, and the low overall costs represent substantial advantages over existing methods. In future, we will expand the range of assays to other proteins to fully exploit the potential of this versatile technique.
